# Prognostic and clinicopathological significance of Hapto and Gremlin1 expression in extrahepatic cholangiocarcinoma

**DOI:** 10.7150/jca.36886

**Published:** 2020-01-01

**Authors:** Zhengchun Wu, Rushi Liu, Xiongying Miao, Daiqiang Li, Qiong Zou, Yuan Yuan, Zhulin Yang

**Affiliations:** 1 Department of General Surgery, Second Xiangya Hospital, Central South University, Changsha, Hunan 410011, China.; 2 School of Medicine. Hunan Normal University, Changsha, Hunan 410013, China.; 3 Department of Pathology, Second Xiangya Hospital, Central South University, Changsha, Hunan 410011, China.; 4 Department of Pathology, Third Xiangya Hospital, Central South University, Changsha, Hunan 410013, China.

**Keywords:** extrahepatic cholangiocarcinoma, biliary tract adenoma, Hapto, Gremlin1, immunohistochemistry

## Abstract

**Background:** Some studies have demonstrated that Hapto and Gremlin1 play an important biological role in many neoplasms. However, the role of Hapto and Gremlin1 in extrahepatic cholangiocarcinoma (ECC) remains to be revealed. Thus, this study investigated the prognostic and clinicopathological significance of Hapto and Gremlin1 expression in ECC.

**Methods:** We examined Hapto and Gremlin1 expression in 100 ECC, 30 peritumoral tissues, 10 adenoma and 15 normal biliary tract tissues using EnVision immunohistochemistry. The relationship between Hapto and Gremlin 1 expression and clinicopathological parameters was evaluated using the χ^2^ test or Fisher's exact test. The overall survival of patients was analyzed using Kaplan-Meier univariate survival analysis and log-rank tests.

**Results:** Hapto and Gremlin1 proteins were overexpressed in ECC compared to peritumoral tissues, adenoma, and normal biliary tract (*P*<0.05 or *P*<0.01). The positive rate of Hapto and Gremlin1 expression was significantly higher in cases with poor differentiation, lymph node metastasis, invasion of surrounding tissues and organs, a tumor-node-metastasis (TNM) stage of III or IV and no resection. Kaplan-Meier survival analysis showed that ECC patients with positive Hapto and/or Gremlin1 expression survived significantly shorter than patients with negative Hapto and/or Gremlin1 expression. Cox multivariate analysis revealed that positive Hapto and Gremlin1 expression were independent poor prognostic factors in ECC patients.

**Conclusion:** The present study indicated that positive Hapto and/or Gremlin1 expression are closely associated with the pathogenesis, clinical, pathological and biological behaviors, and poor prognosis in ECC.

## Introduction

Cholangiocarcinoma (CCA), a tumor originating in the biliary tree epithelium, is the second most common primary hepatic malignancy following hepatocellular carcinoma, and accounts for approximately 3% of gastrointestinal malignancies 1. CCA is an aggressive cancer with high mortality and poor prognosis. The 5-year survival rate of CCA is only 10%, and the confirmed CCA patients have a median survival of 24 months 2. There are various identified risk factors for CCA tumorigenesis, such as primary sclerosing cholangitis, primary or secondary biliary cirrhosis, biliary malformations/choledochal cysts, cholelithiasis/ choledocholithiasis, liver flukes, cirrhosis, alcoholic liver disease, and type Ⅱ diabetes 3. According to tumor occurring anatomical location, CCA is divided into intrahepatic cholangiocarcinoma (ICC, 10%) and extrahepatic cholangiocarcinoma (ECC, 90%) 1, 4. ECC is further distinguished into perihilar (Klatskin) tumors involving the hepatic duct bifurcation (50%) and distal duct tumor (40%) 1, 4. ICC is subdivided into 5 subtypes based on the Bismuth-Corlette classification. Clinical manifestations of CCA vary from tumor location. The most frequent clinical symptom of ECC is painless obstructive jaundice 5. The diagnosis of ECC is made by clinical presentation, imaging techniques including ultrasonography, computed tomography (CT) and magnetic resonance imaging (MRI), and tissue sampling during endoscopic retrograde cholangiopancreatography (ERCP) 1. ECC consists of four histologic grades: well-differentiated, moderately differentiated, poorly differentiated, and undifferentiated, and the majority of ECC is well-differentiated adenocarcinomas 6. Surgery is the only curative treatment for ECC patients. Due to the lack of early clinical manifestations and reliable diagnostic biomarkers, the majority of ECC patients are diagnosed at late clinical stages and lost the opportunity to receive curative surgery 7. Therefore, it is imperative to discover new specific diagnostic biomarkers for the early diagnosis of ECC.

Haptoglobin (Hapto), an acute phase protein in human plasma, is mainly produced by liver 8. Among the plasma proteins, the Hapto content is next to albumin and immunoglobin. During acute inflammation, Hapto concentration is obviously increased in response to inflammatory cytokines (such as interleukin-6) and glucocorticosteroids 8. Hapto with hemoglobin-binding capacity has strong anti-oxidant and anti-inflammatory functions 8, 9. The human gene encoding Hapto is located on chromosome 16q22 and consists of 2 alleles, Hapto 1 and Hapto 2. Accordingly, human Hapto exists as three major phenotypes, Hapto 1-1, Hapto 2-1 and Hapto 2-2. Previous studies reported that Hapto may play an important role in anti-infection and anti-cancer by influencing iron metabolism of invading pathogens and tumor cells, and by affecting angiogenesis and other inflammatory processes 9, 10. Recently, several studies have revealed that the elevated cellular expressive level or serum contain of Hapto is associated with various cancers, such as lung cancer, breast cancer, hepatocellular carcinoma, gastric cancer, colorectal cancer, prostate cancer, bladder cancer and ovarian cancer 11-19. Thus, Hapto might be an important marker in reflecting the malignant degrees, biological behaviors, and prognosis of some malignant lesions.

Gremlin is a member of the Dan family of secreted glycosylated proteins 20. Gremlin contains a highly conserved cysteine knot domain which is also present in transforming growth factor-β (TGF-β) superfamily, platelet-derived growth factor (PDGF), nerve growth factor, and other secreted proteins 21. Gremlin consists of three subtypes, Gremlin1, Gremlin2, and Gremlin3. Gremlin1, a secreted glycoprotein, antagonizes bone morphogenetic proteins (BMPs) 2, 4, and 7, and inhibits transforming growth factor-β signaling, by blocking the interaction of these ligands with their receptors 22, 23. Gremlin1 is also a novel proangiogenic factor that induces angiogenesis in a BMP-independent manner by activating vascular endothelial growth factors receptor-2 (VEGFR2)-dependent angiogenic responses 24, 25. In addition, Gremlin1 plays an important role in cell and tissue differentiation by binding to BMPs and is linked to various types of diseases. Previous studies demonstrated that Gremlin1 is overexpressed in various types of human cancers 26. It has been reported that Gremlin1 is associated with tumorigenesis of several cancers, including oral cancer, glioma, colorectal cancer, hepatocellular cancer and renal cancer 22, 27-33.

Given that the role of Hapto and Gremlin1 in ECC remains to be elucidated, we evaluated Hapto and Gremlin1 expression in surgically resected specimens, including ECC, pericancerous tissues, adenoma, and normal biliary tract, using immunohistochemistry. The clinicopathological significance and prognostic values of Hapto and Gremlin1 expression were analyzed.

## Material and methods

### Case selection

The present retrospective study was approved by the Ethics Committee for Human Research, Central South University, and was conducted according to the approved guidelines. The included patients were histologically diagnosed by two pathologists, and the included ECC patients never received chemotherapy or radiation therapy preoperatively and postoperatively. The patients who received chemotherapy or radiation therapy were excluded. We obtained 100 ECC, 30 peritumoral tissues, 10 biliary tract adenoma, and 15 normal biliary tract tissues at the Second and Third Xiangya Hospitals, Central South University from January 2001 to December 2013. Tumors were restaged according to the 7th TNM Classification of Malignant Tumors and classified following the World Health Organization (WHO) tumor classification system. Tumor differentiated degrees were defined according to the World Health Organization criteria (well-differentiated, moderately differentiated and poorly differentiated).

Survival data for the 100 patients with ECC was obtained through letters and/or telephone calls. The follow-up period was 30 months, and patients who survived longer than 30 months were included in the analysis as censored cases.

We collected 30 pericancerous tissues from the ECC patients undergone radical resection, who consist of 20 male (66.6%) and 10 female (33.3%), and their ages ranged from 35 to 72 (48.5 ± 9.2) years. The pathological examination showed that the 30 pericancerous tissues included 12 normal tissues, 8 mild dysplasia, 6 moderately dysplasia and 4 severe dysplasia. We collected 10 biliary tract adenoma tissues from operative resected specimens. The 10 patients with biliary tract adenoma included 6 male (60.0%) and 4 female (40%), and their ages varied from 33 to 70 (46.7 ± 10.2) years. The pathological examination showed that these biliary tract adenoma included 6 normal tissues, 2 mild dysplasia, and 2 moderate to severe dysplasia. Fifteen normal biliary tract tissues were collected from contributors of liver transplantation, and were confirmed by pathological examination.

All tissues were treated with 4% formaldehyde for 24 to 48 hours and were then routinely embedded in paraffin.

### Immunohistochemistry

Rabbit anti-human Hapto and Gremlin1 polyclonal antibody were purchased from Dako Corporation (Carpentaria, CA, USA). EnVisionTM Detection Kit was purchased from Dako Laboratories (CA, USA). Positive controls were provided with the EnVisionTM Detection Kit. EnVision immunohistochemistry of Hapto and Gremlin1 was performed following the user manual. Briefly, 4 μM-thick sections were cut from paraffin-embedded tissues. The sections were deparaffinized and then incubated with 3% H_2_O_2_ in the dark for 15 min. Heat-induced epitope retrieval was conducted with sodium citrate buffer (10 mM Sodium citrate, 0.05%Tween 20, pH 6.0) at 96℃ for 30 min. The sections were incubated with rabbit anti-human Hapto and Gremlin1 primary antibody (1:100 dilution) for 2 hours after they were soaked in PBS for 3 × 5 min. The sections were incubated with several drops of Solution A (ChemMateTMEnVison+/HRP) for 30 min followed by DAB staining and haematoxylin counter-staining. The sections were dehydrated, soaked in xylene, and mounted with neutral balsam.

Under a magnification of 200 times, 10 random fields per section were viewed by 2 observers independently. The percent of positively stained cells relative to the total number of cells in each field was determined, and the average percentage per case was calculated from the ten fields. The staining intensity was rated on a scale of 1 to 3. A score of 1 represented no positive staining or uncertainly weak staining; a score of 2 represented weak to moderate staining; and a score of 3 represented moderate to strong staining. A case was determined as positive Hapto or Gremlin1 expression when the average percentage of positively stained cells was ≥10% and staining intensity was ≥2. Few cases whose percentage of positive staining was 5% to 10% and staining intensity was 3 were also regarded as positive.

### Statistical analysis

Data was analyzed using the SPSS 17.0 (statistical package for the Social Sciences, Version 17.0). The relationship between Hapto and Gremlin1 expression and clinicopathological parameters was analyzed using the χ^2^ test or Fisher's exact test. The overall survival of patients with ECC was analyzed using Kaplan-Meier univariate survival analysis and log-rank tests. Multivariate analysis was performed with the Cox proportional hazards model and the 95% confidence interval was calculated. A *p* < 0.05 was considered statistically significant.

## Results

### Characteristics of patients

Clinicopathological data for ECC is summarized in Table 2. Among the 100 ECC samples, 61 were from male patients and 39 were from female patients (M/F = 1.56). The 100 ECC patient ages ranged from 35 to 80 (58.8 ± 10.2) years. Of the 100 ECCs, 31 were well-differentiated (31.0%), 34 were moderately differentiated (34.0%) and 35 were poorly differentiated (35.0%). Among the 100 patients with ECC, invasion of surrounding tissues/organs was found in 67 (67.0%); 38 (38.0%) had regional lymph node metastasis; and 31 (31.0%) had gallstones. According to TNM staging, 35 of the 100 ECCs were in stage Ⅰ + Ⅱ, 38 were in stage Ⅲ and 27 were in stage Ⅳ. Among the 100 patients, 54 (54.0%) received radical resection, 36 (36.0%) received palliative resection and only 10 (10.0%) received biopsy.

### Hapto and Gremlin 1 protein expression in ECC, peritumoral tissues, adenoma, and normal tissues

**Figure 1:**
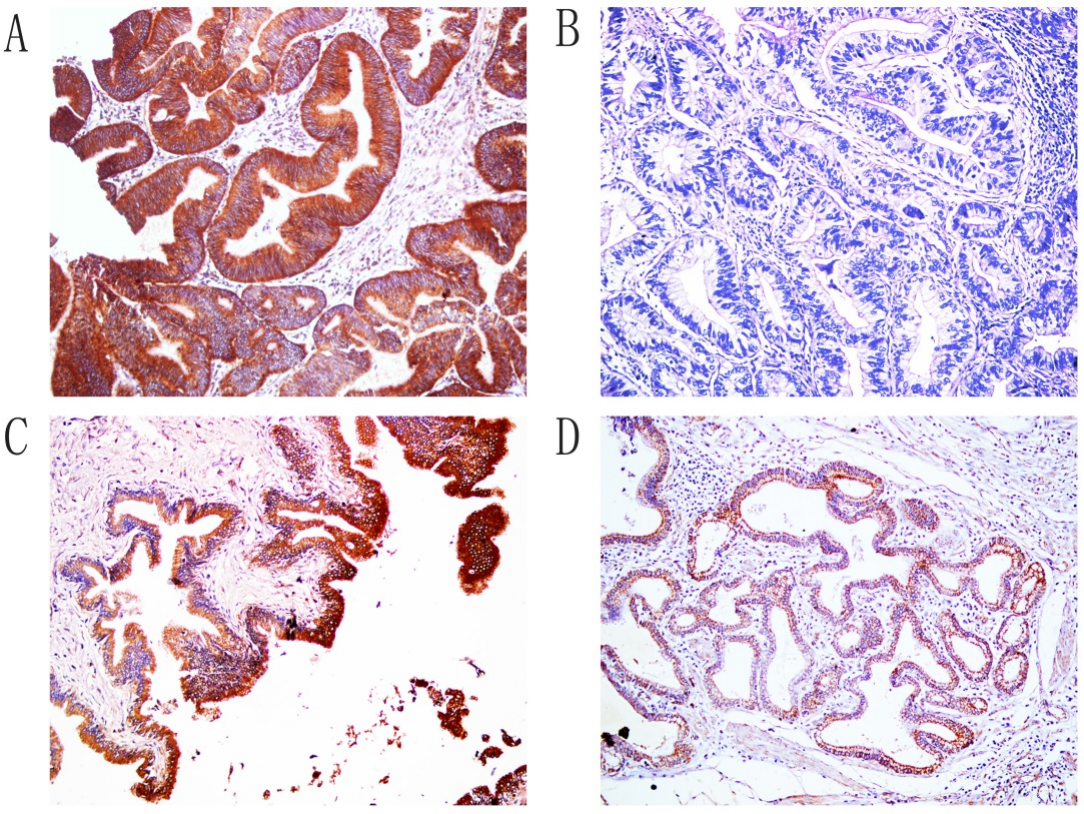
Immunohistochemical staining of Hapto, ×200. A. Positive expression of Hapto, moderately-differentiated ECC. B. Negative expression of Hapto, well differentiated ECC. C. The positive expression of Hapto, pericancerous tissues. D. The positive expression of Hapto, adenoma.

**Figure 2:**
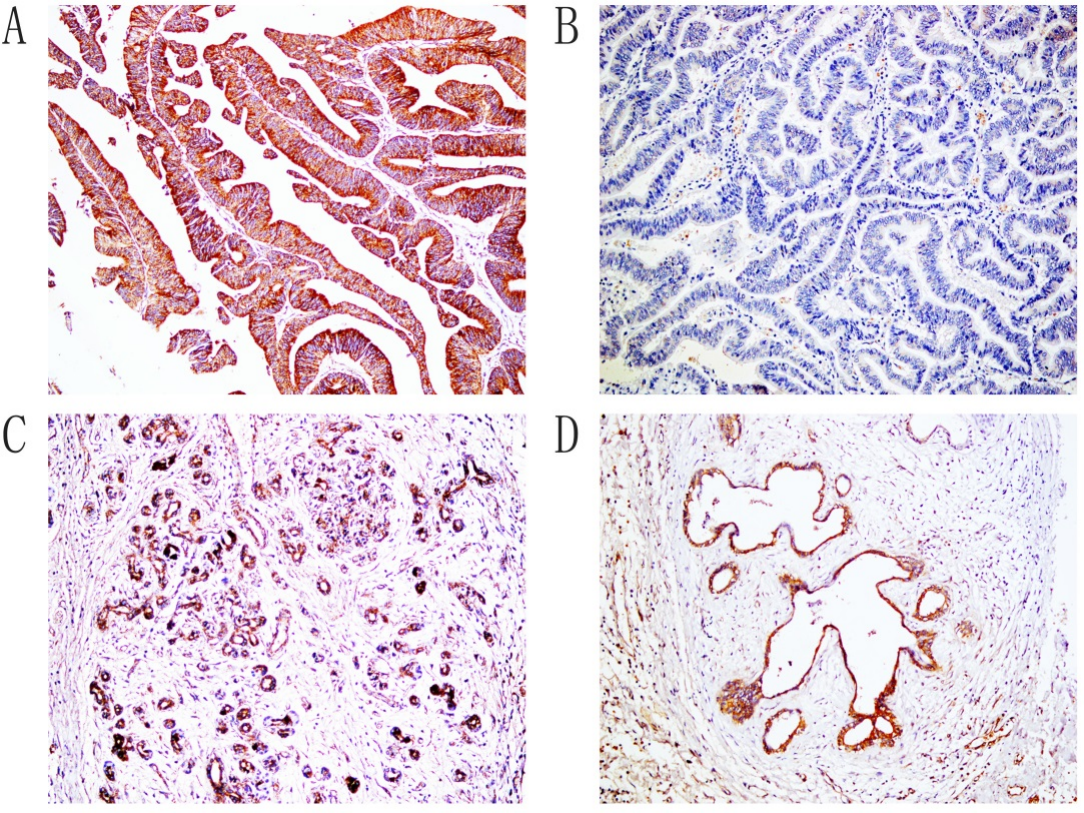
Immunohistochemical staining of Gremlin1, ×200. A. Positive expression of Gremlin1, moderately differentiated ECC. B. Negative expression of Gremlin1, well differentiated ECC. C. The positive expression of Gremlin1, pericancerous tissues. D. The positive expression of Gremlin1, adenoma.

Immunohistochemical staining showed that positive Hapto and Gremlin1 expression were located in the cytoplasm (Figure 1 and 2). In the 100 ECCs, 60 and 53 were Hapto (60.0%) and Gremlin1 (53.0%) positive, respectively. In the 30 peritumoral tissues, 8 and 9 were Hapto (26.7%) and Gremlin1 (30.0%) positive, respectively. In 10 adenoma, 2 and 1 were Hapto (20.0%) and Gremlin1 (10.0%) positive, respectively. In all 15 normal tissues, Hapto and Gremlin1 expression were negative. The positive rate of Hapto or Gremlin1 was significantly higher in extrahepatic cholangiocarcinoma than in peritumoral, adenoma, and normal tissues (*P*<0.05 or *P* <0.01) (Table 1). Peritumoral tissues and adenoma with positive Hapto and/or Gremlin1 expression exhibited moderate to severe dysplasia (Table 1).

### Hapto and Gremlin1 protein expression were associated with clinicopathological characteristics of ECC

As shown in Table 2, positive rates of Hapto and Gremlin 1 expression were significantly lower in cases with well differentiation, no lymph node metastasis, no invasion of surrounding tissues and organs, a TNM stage of I + II, and radical resection compared to cases with poor differentiation, lymph node metastasis, invasion of surrounding tissues and organs, a TNM stage of III or IV, and no resection (biopsy only) (*P* < 0.05 or *P* < 0.01). The positive rate of Hapto expression was significantly lower in cases with tumor diameter >3cm than that in ones with tumor diameter <3cm (*P* < 0.05). The expression of Hapto and Gremlin1 exhibited no significant association with sex, age, and tumor site (*P* > 0.05). Among the 60 cases with positive Hapto expression, 40 cases had positive Gremlin1 expression. Among the 40 cases with negative Hapto expression, 27 cases had negative Gremlin1 expression. The expression of Hapto was positively correlated with Gremlin1 in ECC (χ^2^=11.247, *P* = 0.001).

### Hapto and Gremlin1 protein expression correlated with overall survival in patients with ECC

**Figure 3:**
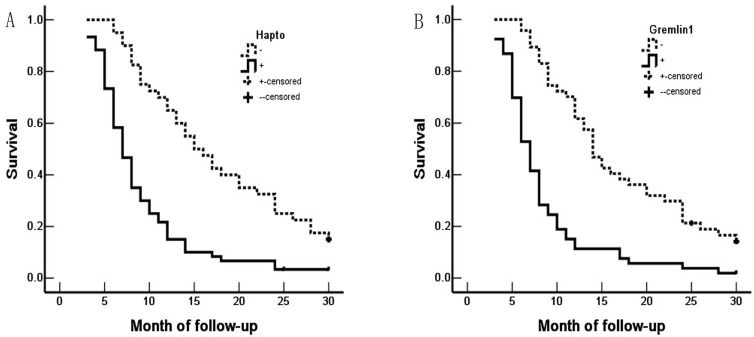
Hapto and Gremlin1 expression and survival in patients with ECC. A. Kaplan-Meier plots of overall survival in patients with Hapto-positive and -negative tumors. B. Kaplan-Meier plots of overall survival in patients with Gremlin1-positive and -negative tumors.

**Figure 4:**
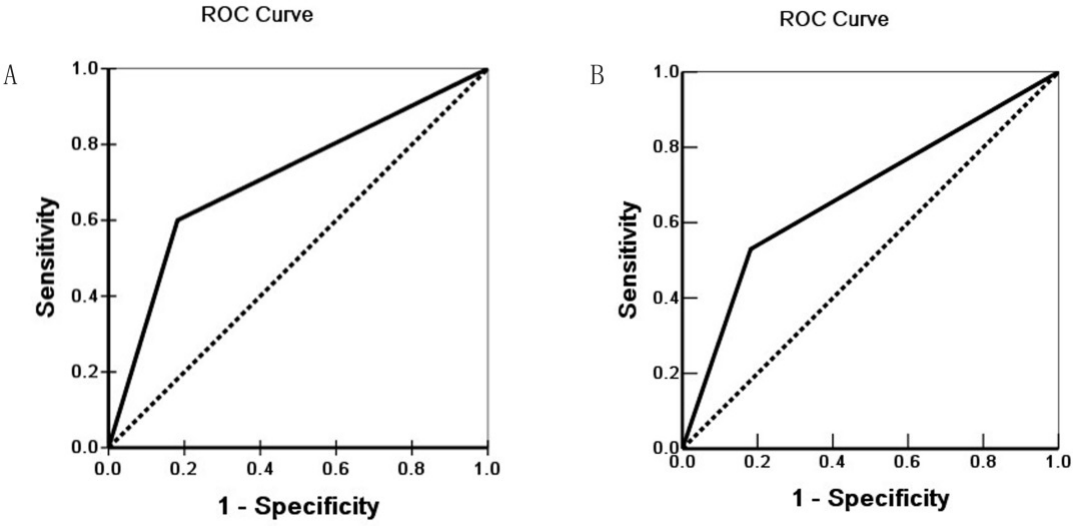
Receiver operating characteristic (ROC) of Diagonal segments are produced by ties of Hapto (A) and Gremlin1 (B).

Survival information of the 100 patients was collected. Of 100 ECC patients, 59 patients died within 12 months, 24 patients died within 24 months, 9 patients died within 30 months, and patients (8 cases) who survived longer than 30 months were included in the analysis as censored cases. Our results revealed that the differentiation, lymph node metastasis, invasion of surrounding tissues and organs, TNM stage and surgical procedure were significantly associated with the average overall survival time of patients with ECC (*P* < 0.05 or *P* <0.01) (Table 3). Kaplan-Meier survival curves demonstrated that average overall survival time for patients with Hapto or Gremlin1 positive expression was significantly lower than those with negative Hapto or Gremlin1 expression (*P* =0.000) (Figure 3). Cox multivariate analysis showed that poor differentiation, lymph node metastasis, invasion, high TNM stage (III or IV) and no resection (only biopsy) negatively correlated with overall survival and positively correlated with mortality. Positive Hapto and/or Gremlin1 expression was negatively correlated with overall survival and positively correlated with mortality. Both Hapto and Gremlin1 positive expression were independent prognostic factors (Table 4). Finally, we calculated the area under the curve value for Hapto (AUC = 0.709, 95%CI: 0.625-0.793), or Gremlin1 (AUC = 0.674, 95%CI: 0.588-0.760), respectively (Figure 4).

## Discussion

The expression of Hapto and Gremlin1 have been associated with the progression and prognosis of a variety of tumors, but their expression in ECC have not been previously reported and their biological roles in ECC remain to be identified. Thus, we investigated Hapto and Gremlin1 protein expression in ECC, peritumoral tissues, adenoma, and normal biliary tract using immunohistochemistry, and analyzed their clinicopathological significance in this study. We found that a significant increase in Hapto and Gremlin1 expression in ECC was observed, compared with non-tumor tissues. Additionally, positive Hapto and Gremlin1 expression were associated with poor differentiation, advanced TNM stages, invasion, metastasis and poor prognosis of ECC. Thus, these results indicated that Hapto and Gremlin1 may play an unneglected role in the tumorigenesis of ECC.

Isoforms of Hapto were markedly differentially expressed protein spots. Hapto is involved in several biological processes, including hemoglobin (Hb) scavenging, inflammatory responses, immune suppression and angiogenesis^34^. In addition to being mainly produced and secreted by liver cells, Hapto is also produced by intestinal, seminiferous and endometrial epithelia^35^. Moreover, it has been reported that Hapto is produced by various types of cancer cells, such as malignant ovarian epithelium, renal cell carcinoma and hepatocellular carcinoma (HCC) cells^36^. Increased serum Hapto which is mainly triggered by interleukins such as IL-6 and TNF-α, is frequently reported in cancer and is thought to be a marker of inflammation and tissue damage 37. Increasing evidence supports the use of serum Hapto (either it's level or modifications) as a biomarker for cancer diagnosis regardless of cancer type. Hapto has also been used to study various liver diseases, including HBV and HCC. Both the amount and oligosaccharide moiety of Hapto change in liver diseases. It has been reported that the Hapto serum level changes significantly in cirrhosis and HCC patients^38^. Pirincci N et al found that the serum haptoglobin levels in the patients with bladder cancer were significantly higher compared with healthy controls^18^. Consistent with this previous study, we found that Hapto expression in ECC was obviously higher than in non-tumor tissues. Previous studies reported that serum Hapto can predict colorectal cancer hepatic metastasis and Hapto promotes colorectal cancer cell invasion^39^, and high serum haptoglobin levels are closely associated with distant metastasis, lymphovascular involvement, lymph node metastasis and increasing tumor burden in bladder cancer^18^. Similarly, our study showed that the positive rate of Hapto expression was higher in patients with poor differentiation, lymph node metastasis, invasion of surrounding tissues/organs, and a TNM stage of III or IV and no resection. In addition, several tumor types express Hapto, and its expression correlates with poor prognosis in lung cancer, breast cancer, hepatocellular carcinoma, gastric cancer, colorectal cancer, prostate cancer, bladder cancer and ovarian cancer^11^-^19^. In this study, we found similar results that positive Hapto expression negatively correlated with overall survival and positively correlated with mortality. Thus, Hapto may be an important marker in reflecting the malignant degrees, biological behaviors, and prognosis of ECC, and play a key biological role in the occurrence and development of ECC, which requires further identified.

Gremlin 1 is a secreted protein and consists of three isoforms including isoforms 1, 2 and 3. The gene encoding human Gremlin 1 maps to chromosome 15q13-q15^40^. As a BMP antagonist, Gremlin 1 can specifically bind to BMP-2, BMP-4, and BMP-7, and thus block their interaction with receptors^38^. In addition, Gremlin 1 is involved in angiogenesis by binding to VEGFR2. Accumulating evidence shows that Gremlin 1 is associated with tumor. It has been reported that Gremlin 1 can drive cell migration, proliferation, and invasion in various cancer cell lines^41^. Some studies have demonstrated that Gremlin 1 is overexpressed in some human cancers including colorectal cancer, pancreatic cancer, lung cancer, ovary cancer, renal cancer, breast cancer, cervical cancer, glioblastoma, hepatocellular carcinoma and diffuse B-cell lymphoma^33^, ^42^. In agreement with these previous studies, we found that positive rate of Gremlin 1 was significantly higher in ECC than in peritumoral, adenoma, and normal tissues in this study, which indicated that Gremlin 1 may be related to carcinogenesis of ECC. Additionally, many reports have revealed that Gremlin 1 plays an essential role in a variety of cancers development and progression, such as colorectal cancer, pancreatic cancer, and renal cancer^22^, ^40^, ^43^. Yu et al demonstrated that Gremlin 1 positive expression is associated with poor clinical prognosis and TNM stage in pancreatic ductal cancer, and Gremlin 1 can induce pancreatic cancer cell invasion, migration and epithelial to mesenchymal transition (EMT)^40^. Liu et al reported that knockdown Gremlin 1 inhibited cell proliferation, angiogenesis, and EMT in colon cancer^43^. Gremlin 1 is involved in regulating colorectal cancer progression^30^, and Davis et al reported that the colorectal cancer patients with high Gremlin 1 expression have significantly shorter disease-free survival compared with the patients with low Gremlin 1 expression^44^. Moreover, Stao et al also found similar results that patients with a high Gremlin 1 mRNA expression have significantly poor prognosis of progression in cervical cancer^26^. In this study, our data showed that Gremlin 1 positive expression was positively associated with poor differentiation, advanced TNM stages, invasion, metastasis, and poor prognosis of ECC, which is consistent with previous reports. Thus, our results indicated that Gremlin 1 may play an important biological role in ECC, which needs further investigation to identify its specific mechanism.

In the present study, the percentage of cases with positive Hapto and Gremlin1 over-expression was significantly higher in ECC patients with poor differentiation, lymph node metastasis, invasion of surrounding tissues/organs, TNM stage III/IV disease and no resection (biopsy only) than in patients with well differentiation, no lymph node metastasis, no invasion of surrounding tissues/organs, TNM stage I/II disease and radical resection (*P*<0.05 or *P*<0.01). In biliary tract epithelia in pericancerous tissues and adenoma tissues with positive Hapto and Gremlin1 protein expression exhibited moderate to severe dysplasia. Kaplan-Meier survival analysis showed that ECC patients with positive Hapto and/or Gremlin1 expression survived significantly shorter than patients with negative Hapto and/or Gremlin1 expression. Cox multivariate analysis suggested that positive Hapto or Gremlin1 expression is an independent prognostic factor for poor prognosis in patients with ECC. The AUC for Hapto and Gremlin1 showed that they might play an important role in carcinogenesis, progression and early finding or prevention of ECC.

In conclusion, Hapto and Gremlin1 are involved in the tumorigenesis and progression of ECC, and positive Hapto and Gremlin1 expression were associated with poor prognosis in patients with ECC.

## Figures and Tables

**Table 1:** Comparison of Hapto and Gremlin1 expression in normal tissue, adenoma, peritumoral tissue and ECC.

Tissue type	Case No.	Hapto positive (%)	Gremlin1 positive (%)
ECC	100	60 (60.0)	53 (53.0)
Peritumoral tissues	30	8 (26.7)* *	9 (30.0)*
adenoma	10	2 (20.0)*	1 (10.0)*
Normal tissues	15	0 (0.0)**	0 (0.0)**

Abbreviation: ECC, extrahepatic cholangiocarcinoma; Compared to ECC: * *P* < 0.05; ** *P* < 0.01.

**Table 2:** Correlations of Hapto and Gremlin1 protein expression with the clinicopathological characteristics of ECC.

CPC	Number of Patients (N)	Hapto				Gremlin1		
		Pos No. (%)	χ^2^	*P*		Pos No. (%)	χ^2^	*P*
**Age (year)**								
≤45 years	17	9 (52.9)	0.425	0.514		7 (41.2)	1.149	0.284
>45 years	83	51 (51.4)				46 (55.4)		
**Sex**								
Male	61	38 (62.3)	0.343	0.558		32 (52.5)	0.018	0.892
Female	39	22 (56.4)				21 (53.8)		
**Differentiation**								
Well	31	11 (35.5)	12.363	0.002		9 (29.0)	11.019	0.004
Moderately	34	22 (64.7)				20 (58.8)		
Poorly	35	27 (77.7)				24 (68.6)		
**Tumor size**								
≤3cm	62	42 (67.7)	4.075	0.044		34 (54.8)	0.221	0.638
>3cm	38	18 (47.4)				19 (50.0)		
**Tumor site**								
Hilar site	27	17 (63.0)	0.592	0.744		16 (59.3)	0.583	0.747
Hepatic duct	4	3 (75.0.0)				2 (50.0)		
Distal duct	69	40 (58.0)				35 (50.7)		
**Bile stone**								
No	69	36 (52.2)	5.680	0.017		32 (46.4)	3.920	0.048
Yes	31	24 (77.4)				21 (67.7)		
**Lymph node metastasis**								
No	62	27 (43.5)	18.400	0.000		23 (37.1)	16.566	0.000
Yes	38	33 (86.8)				30 (78.9)		
**Invasion of surrounding tissues/organs**								
No	33	13 (39.4)	8.714	0.003		10 (30.3)	10.186	0.001
Yes	67	47 (70.1)				43 (64.2)		
**TNM stage**								
I + II	35	12 (34.3)	15.957	0.000		9 (25.7)	24.424	0.000
III	38	26 (68.4)				20 (52.6)		
IV	27	22 (81.5)				24 (88.9)		
**Surgery**								
Radical	54	22 (40.7)	18.179	0.000		22 (40.7)	7.898	0.019
Palliative	36	30 (83.3)				23 (63.9)		
Biopsy	10	8 (80.0)				8 (80.0)		

Abbreviation: CPC, Clinicopathological characteristics; Pos No., Positive Number.

**Table 3:** Correlations of clinicopathological characteristics, Hapto and Gremlin1 expression with the mean survival in patients with ECC.

Group	Number of patients (N)	Mean survival (month)	χ^2^	*P*
**Sex**				
Male	61	12.67 (3-30)	0.001	0.980
Female	39	12.59 (4-30)		
**Age (year)**				
≤45	17	13.82 (3-30)	0.667	0.414
>45	83	12.10 (3-30)		
**Differentiation**				
Well	31	18.46 (5-30)		
Moderately	34	11.41 (3-30)	27.655	0.000
Poorly	35	7.97 (3-30)		
**Tumor size**				
≤3cm	62	12.62 (3-30)	0.235	0.628
>3cm	38	12.03 (5-30)		
**TNM stage**				
I + II	35	18.57 (7-30)	57.569	0.000
III	38	11.05 (3-30)		
IV	27	6.26 (3-13)		
**Lymph node metastasis**				
No	62	15.52 (4-30)	39.001	0.000
Yes	38	7.18 (3-25)		
**Invasion of surrounding tissues/organs**				
No	33	17.52 (4-30)	17.399	0.000
Yes	67	9.87 (3-30)		
**Surgery**				
Radical	54	16.62 (3-30)	48.388	0.000
Palliative	36	7.58 (4-24)		
Biopsy	10	6.90 (3-14)		
**Hapto**				
-	40	17.50 (6-30)	25.589	0.000
+	60	9.00 (3-30)		
**Gremlin1**				
-	47	16.82 (6-30)	30.155	0.000
+	53	8.47 (3-30)		

Abbreviation: -, negative expression; +, positive expression.

**Table 4:** Multivariate Cox regression analysis of survival rate in patients with ECC and Hapto and Gremlin1 expression.

Groups	Factors	B	SE	wald	*P*	RR	95% CI	
							Lower	Upper
Differentiated degree	Well/moderately/poorly	.482	.160	9.075	.003	1.619	1.183	2.216
Tumor size	<3 cm/3∼5 cm/>5 cm	.513	.248	4.279	.039	1.670	1.027	2.716
Lymph node metastasis	No/Yes	.797	.321	6.165	.013	2.219	1.183	4.163
Invasion of surrounding tissues/organs	No/Yes	.818	.370	4.888	.027	2.266	1.097	4.680
TNM stage	I/II/III/IV	.478	.185	6.676	.010	1.613	1.122	2.318
Surgery	Radical/Palliative/Biopsy	.818	.370	4.888	.027	2.266	1.097	4.680
Hapto	-/+	.596	.271	4.837	.028	1.815	1.067	3.087
Gremlin1	-/+	.778	.284	7.505	.006	2.177	1.248	3.799

Abbreviations: -, negative expression; +, positive expression; RR, relative risk, CI, confidence interval.

## Abbreviations

CCA: Cholangiocarcinoma
ECC: extrahepatic cholangiocarcinoma
ICC: intrahepatic cholangiocarcinoma
CT: computed tomography
MRI: magnetic resonance imaging
ERCP: endoscopic retrograde cholangiopancreatography
Hapto: Haptoglobin
TGF-β: transforming growth factor-β
PDGF: platelet-derived growth factor
BMP: bone morphogenetic protein
WHO: World Health Organization
TNM: tumor-node- metastasis
AUC: The area under the curve
CI: confidence interval
Hb: hemoglobin
HCC: hepatocellular carcinoma
IL-6: interleukin-6
TNF-α: tumor necrosis factor-α
HBV: hepatitis B virus
VEGFR2: vascular endothelial growth factors receptor-2
EMT: epithelial to mesenchymal transition
ROC: Receiver operating characteristic
CPC: Clinicopathological characteristics
RR: relative risk
